# Dual-modality rigid endoscope for photoacoustic imaging and white light videoscopy

**DOI:** 10.1117/1.JBO.29.2.020502

**Published:** 2024-02-15

**Authors:** Rehman Ansari, Edward Zhang, Paul Beard

**Affiliations:** a UCL, Department of Medical Physics and Biomedical Engineering, London, United Kingdom; b UCL, Wellcome/EPSRC Centre for Interventional and Surgical Sciences, London, United Kingdom

**Keywords:** photoacoustic tomography, photoacoustic endoscopy, white light endoscope, Fabry–Perot ultrasound sensor

## Abstract

**Significance:**

There has been significant interest in the development of miniature photoacoustic imaging probes for a variety of clinical uses, including the *in situ* assessment of tumors and minimally invasive surgical guidance. Most of the previously implemented probes are either side viewing or operate in the optical-resolution microscopy mode in which the imaging depth is limited to ∼1  mm. We describe a forward-viewing photoacoustic probe that operates in tomography mode and simultaneously provides white light video images.

**Aim:**

We aim to develop a dual-modality endoscope capable of performing high-resolution PAT imaging and traditional white light videoscopy simultaneously in the forward-viewing configuration.

**Approach:**

We used a Fabry–Pérot ultrasound sensor that operates in the 1500 to 1600 nm wavelength range and is transparent in the visible and near infrared region (580 to 1250 nm). The FP sensor was optically scanned using a miniature MEMs mirror located at the proximal end of the endoscope, resulting in a system that is sufficiently compact (10 mm outer diameter) and lightweight for practical endoscopic use.

**Results:**

The imaging performance of the endoscope is evaluated, and dual-mode imaging capability is demonstrated using phantoms and abdominal organs of an *ex vivo* mouse including spleen, liver, and kidney.

**Conclusions:**

The proposed endoscope design offers several advantages including the high acoustic sensitivity and wide detection bandwidth of the FP sensor, dual-mode imaging capability, compact footprint, and an all-optical distal end for improved safety. The dual-mode imaging capability also offers the advantage of correlating the tissue surface morphology with the underlying vascular anatomy. Potential applications include the guidance of laparoscopic surgery and other interventional procedures.

## 1 Introduction

Photoacoustic (PA) imaging is an emerging technique capable of visualizing the vasculature deep within the tissue by exploiting intrinsic molecular contrast.^1^^,^^2^ Miniaturized PA probes have many potential applications in intraoperative imaging and minimally invasive surgical guidance. These include the *in situ* assessment of cancerous lesions in the abdominal organs, identification of tumor margins during laparoscopic liver surgery, guidance of needle biopsies, and treatment monitoring of photocoagulation therapy. These applications often require a forward-viewing configuration to visualize the tissue directly ahead of the probe tip. However, the majority of previously reported PA endoscopic probes tend to be side viewing.^3^^–^^7^ Of the few forward-viewing probes that have been demonstrated, almost all employ optical resolution microscopy (OR-PAM)^8^^–^^11^ in which the imaging depth is restricted to <1  mm. For greater penetration depths, widefield photoacoustic tomography (PAT) mode is desirable.^1^ Unlike OR-PAM, which employs a scanning focused excitation laser beam and a single static detector, PAT requires widefield illumination and mapping of the PA wavefield at multiple spatial points. This poses several technical challenges. First, for high resolution three-dimensional (3D) PAT, a high-density two-dimensional (2D) ultrasound array that provides fine spatial sampling (<100  μm) and wide detection bandwidth (tens of MHz) is required but is challenging to achieve with conventional piezoelectric detection technology.^12^ Capacitive micromachined ultrasonic transducers (CMUT) fabrication technology can produce high-density arrays^13^^–^^16^ but lacks the large detection bandwidth for high-resolution PAT imaging. Second, efficient delivery of the excitation light to the tissue requires delivering the widefield illumination through the detector to the tissue. This necessitates a transparent detector array, but most conventional piezoelectric receivers and CMUTs are opaque.

The Fabry–Pérot (FP) ultrasound sensing concept^17^ can address these limitations by providing the requisite wide-bandwidth, fine spatial sampling and an optically transparent sensor head. Miniature, forward-viewing 3D PAT endoscopic probes based on this type of sensor have been demonstrated.^18^^,^^19^ These devices employ a high density coherent fiber bundle to read out the FP sensor, which offers a high degree of miniaturization^19^ and the flexibility^18^ for minimally invasive applications that require tortuous access. In this paper, we describe an endoscopic probe based on a different design. Instead of using a fiber bundle to read out the sensor, a free-space scanning beam delivered via a miniature MEMs scanner and an optical relay is used. This approach avoids the need (i) for a scanner system that can provide sub-micron optical positional accuracy to couple the interrogation laser beam into the cores of the fiber bundle and (ii) to overcome the limitations of commercially available fiber bundles, which tend not to be designed for single-mode operation at the 1500 to 1600 nm FP sensor interrogation wavelength range and can result in additional noise.^20^ Although these challenges are not fundamental limitations, they do make free-space interrogation a more attractive proposition for applications in which a high degree of miniaturization and flexibility of the fiber-bundle based probes are not required. For example, for an application such as guiding laparoscopic surgery of the liver, a rigid probe with an outer diameter in the 10 to 15 mm range (which is similar to that of a rigid laparoscopic ultrasound probe) would be acceptable and amenable to a free-space FP sensor read-out scheme.

A forward-viewing rigid endoscope based on the above free-space read-out design that provides high-resolution 3D PAT imaging is described. An additional novel aspect is that the probe is designed to provide an integrated video rate white light imaging capability that enables the target tissue ahead of the probe tip to be visualized in real time. The dual-modality approach enables a comprehensive *in situ* assessment of tissue as it enables both visual inspection of the tissue surface and 3D imaging of the subsurface vascular anatomy.

## 2 Experimental Setup

**Fig. 1:**
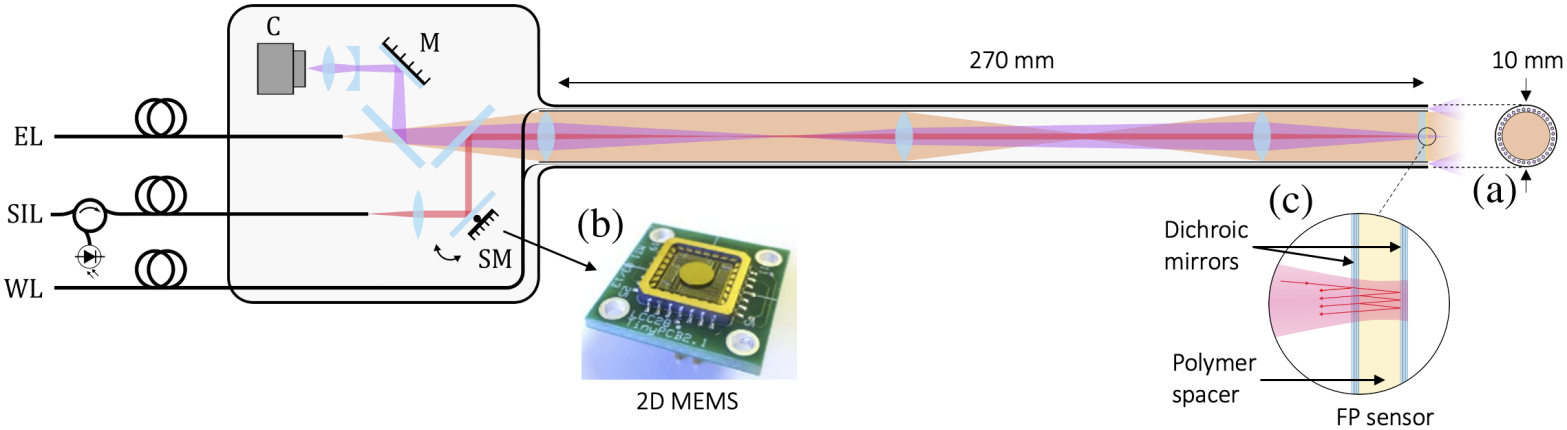
Schematic of the dual-modality PA endoscope. EL, excitation laser; SIL, sensor interrogation laser; WL, constant intensity white light source; SM, 2D MEMS scanning mirror; C, CMOS camera. (a) Cross-section of the endoscope tube, which shows white light illumination fibers. (b) Miniature 2D MEMS mirror used to optically scan the FP sensor with an interrogation laser beam. (c) Schematic showing the cross-section of the FP sensor, which comprises a polymer spacer sandwiched between two dichroic mirror coatings.

A schematic of the dual-modality endoscope is shown in Fig. 1. The probe comprises a stainless-steel tube of length 270 mm and external diameter 10 mm with an optically transparent FP ultrasound sensor located at its distal end. To acquire PAT images, pulsed excitation laser light at 750 nm is delivered along the stainless-steel tube and through the FP sensor to illuminate the tissue. The resulting generated PA waves are recorded in 2D by optically scanning the FP sensor with a focused 1550 nm laser beam. A 3D PAT image is then reconstructed from the measured data. To acquire white light images, an array of optical fibers embedded within the wall of the stainless-steel tube is used to deliver widefield visible light to the tissue. The back-scattered light is then transmitted back through the FP sensor, relayed along the length of the tube, and imaged onto a miniature CMOS camera (C) at the proximal end.

The FP ultrasound sensor comprises a thin film multilayer structure consisting of a 25  μm thick Parylene C spacer sandwiched between a pair of dichroic mirrors that acts as an FP etalon. The mirrors are designed for high transmission in the visible and near infrared spectral region (580 to 1250 nm) and high reflectivity in the short-wave spectral region (1500 to 1600 nm). This enables both the 750 nm PA excitation laser light and the white light used to acquire the video image to be delivered through the sensor and on to the tissue sample. At the same time, the mirrors provide the necessarily high reflectivities over the FP sensor interrogation laser wavelength range (1500 to 1600 nm) for the thin film structure to act as an etalon. By tuning the interrogation laser wavelength to the edge of the etalon transfer function,^17^ acoustically induced optical thickness changes in the spacer thickness produce a corresponding reflected power modulation of the interrogation beam, which enables the detection of the PA waves. To record the PA wavefield at different points across the sensor, a miniature two-axis electrostatic MEMs based scanner (Mirrorcle Technologies Inc.) located at the proximal end of the probe is used to scan a focused interrogation beam of a 62.5  μm diameter across its surface via a 4f optical relay system and a scanning lens. The optical relay serves to image the scanning mirror pivot point onto the back focal plane of the scanning lens to enable the FP sensor to be located 27 cm from the MEMs scanner. The interrogation beam is reflected from the FP sensor, back through the relay system and MEMs scanner, and on to a fiber coupled InGaAs photodiode-transimpedance amplifier unit with a 70 MHz bandwidth. The output of the photodiode is connected to a digitizer with an analog bandwidth of 60 MHz to record the detected PA signals. A tunable (660 to 2200 nm) fiber-coupled optical parametric oscillator-based laser system (Innolas GmbH), which provides 7 ns pulses at a 200 Hz pulse repetition frequency (PRF), is used as the PA excitation source. A short-pass dichroic mirror combines the excitation and sensor interrogation beams at the proximal end of the endoscope. The excitation beam then passes through the optical relay and exits the endoscope tip as a near-collimated 7 mm diameter beam. To reconstruct a 3D PAT image from the detected PA signals, a time reversal algorithm^21^ is used.

A constant intensity LED source (Karl Storz Power LED 175) is used to provide the illumination for the white light endoscopy images. The LED output is coupled into a bundle of 128 multi-mode optical fibers (225/200  μm core/cladding diameters; Thorlabs Inc.) that were embedded within the walls of the stainless-steel tube. These fibers illuminate the target tissue at the tip of the endoscope, and the back-scattered light is relayed back through the optical relay system and imaged onto a 40 Hz CMOS camera (Basler acA-1920-40c). A 25 mm fixed focal length camera lens in combination with a −200  mm focal length plano-concave lens is used to set the widefield imaging focus at a distance of 5 mm from the probe tip. The system was designed to provide an overall magnification of 2.5×, a 7 mm diameter field-of-view, and a 5 mm depth of focus.

## 3 Results

The PAT imaging performance of the dual-modality endoscope was evaluated in terms of noise-equivalent-pressure, detection bandwidth, field-of-view, and lateral and axial resolution. The rms NEP was measured at 8100 spatial positions on the FP sensor, and the modal value was 150 Pa over 20 MHz detection bandwidth. The −3  dB detection bandwidth of the FP sensor, which depends on the optical thickness of the polymer spacer, was estimated to be 35 MHz using an experimentally validated numerical frequency response model.^22^ The field-of-view and spatial resolution were evaluated by imaging a multi-layer black polymer ribbon phantom immersed in a scattering 1% intralipid solution. The ribbons were 20  μm thick and sufficiently wide to approximate as an absorbing step in the lateral direction.

**Fig. 2:**
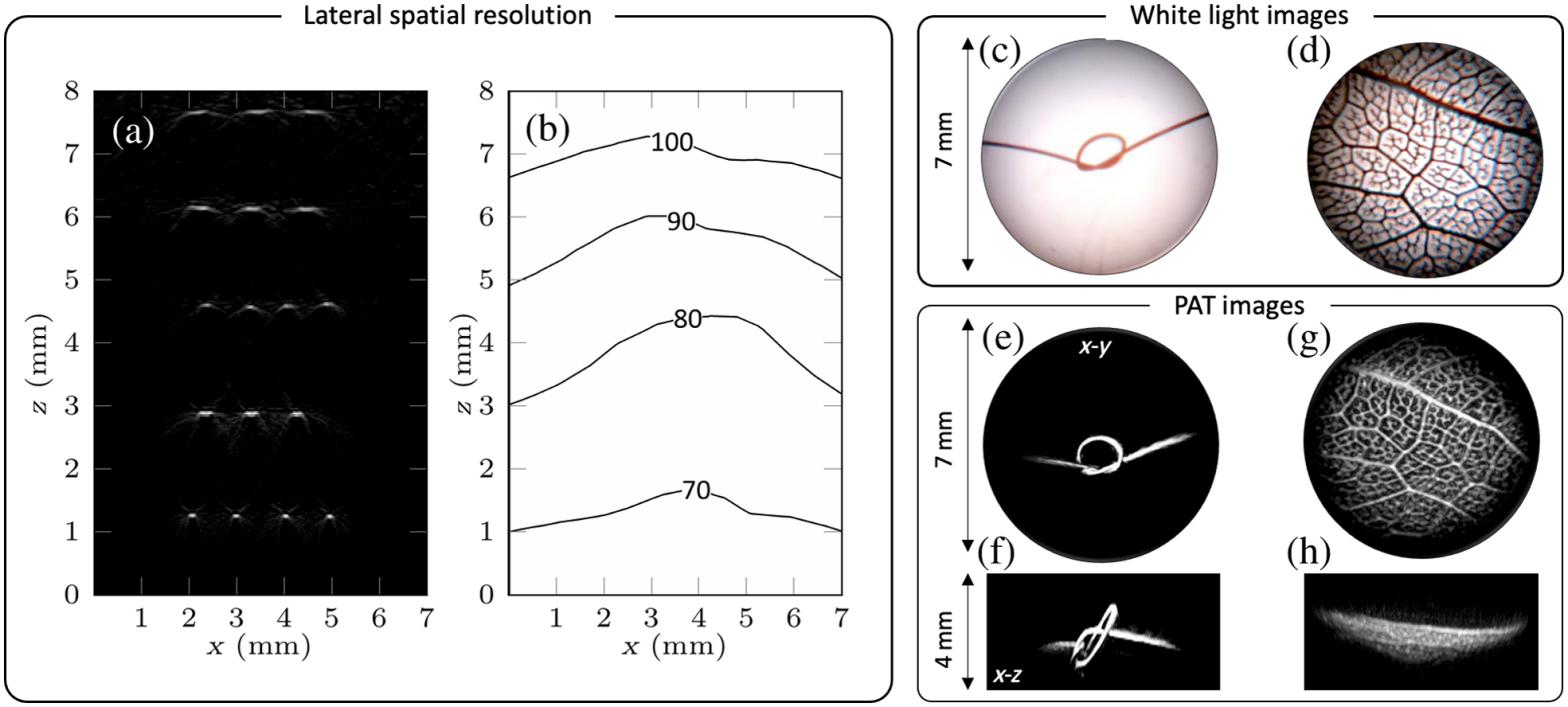
Phantom images: (a) x-z cross-section extracted from the reconstructed 3D PAT image of a multi-layer ribbon phantom that shows a cross-section over the probe field of view. (b) A contour plot showing the variation in the lateral spatial resolution, obtained by evaluating the edge spread function from each ribbon feature. Dual-modality images of arbitrarily shaped phantoms. (c), (d) White light images of a synthetic hair knot and leaf skeleton phantom. (e)–(h) Horizontal and vertical projections from the 3D PAT images of the phantoms.

A 3D PAT image of the phantom was acquired using a 750 nm excitation wavelength, $5\;\mathrm{mJ}/{\mathrm{cm}}^{2}$ fluence, and scanning a 7 mm diameter area in steps of 75  μm with a total acquisition time of 40 s. Figure 2(a) shows a cross-section along the x-z plane from the reconstructed 3D PA image of the ribbon phantom. The edge response function was evaluated for each ribbon cross-section and interpolated over the x-y FOV to estimate the lateral spatial resolution [Fig. 2(b)]. The lateral spatial resolution was 70  μm at a depth of 1.5 mm from the tip of the probe and decreased to 100  μm at a depth of 7 mm. The vertical resolution was estimated at 28  μm based on the bandwidth of the sensor, and it is approximately spatially invariant over the FOV.

The dual-mode imaging capability of the endoscope was first evaluated by imaging arbitrarily shaped absorbing phantoms. Figures 2(c) and 2(d) show the white light images of a synthetic hair knot and leaf skeleton phantom, which were immersed in deionized water. PAT images of these phantoms were acquired using a 750 nm excitation wavelength and $5\;\mathrm{mJ}/{\mathrm{cm}}^{2}$ fluence. The reconstructed PAT images of the phantoms are shown in Figs. 2(e)–2(h), as maximum intensity projections along the horizontal (x-y) and vertical plane (x-z). The structural features of the synthetic hair knot and the intricate veins of the leaf skeleton phantom are accurately reproduced in the PAT images, showing good correspondence with their respective white light endoscopic images.

**Fig. 3:**
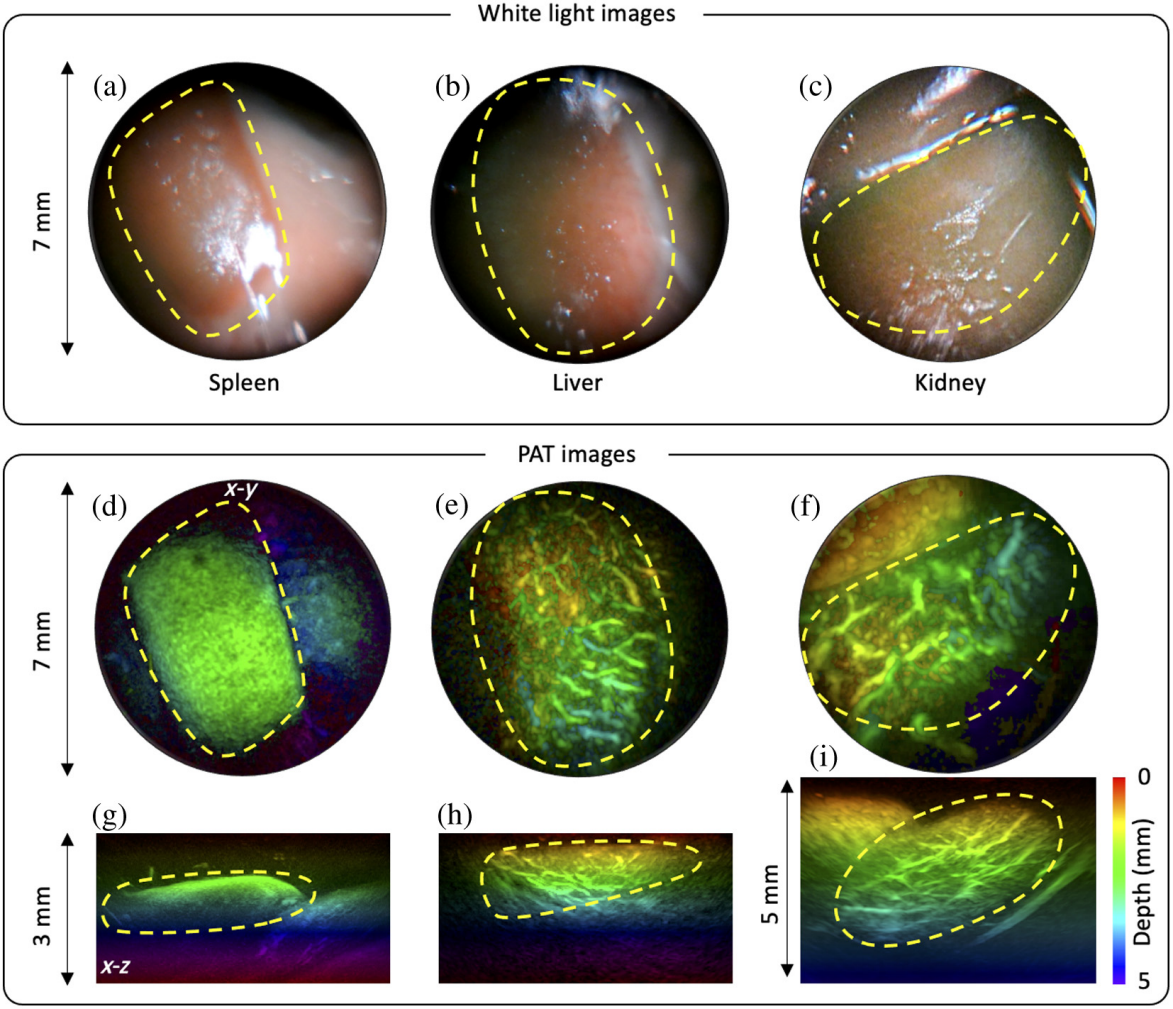
Dual-modality images of *ex vivo* mouse abdominal organs *in situ*. (a)–(c) The top panel shows white light endoscopic images of the spleen, liver, and kidney. (d)–(i) Horizontal and vertical projections from the 3D PAT images showing the underlying vascular anatomy of respective organs. Excitation wavelength: 760 nm. (Video 1, MP4, 704 KB [URL: https://doi.org/10.1117/1.JBO.29.2.020502.s1].)

The dual-modality imaging capability in biological media was demonstrated by examining the abdominal organs of an *ex vivo* mouse, made accessible via a large incision in the abdomen. This is best illustrated in Video 1 (available online), which shows how the white light imaging capability is used to navigate the probe to the organ of interest. Once the probe is correctly positioned, it is gently pressed against the tissue, and a 3D PAT image is acquired. Figure 3 shows selected images from Video 1. The top panel shows the white light images of the spleen, liver, and kidney. The 3D PAT images of the organs from the same locations are shown in the bottom panel of Fig. 3. The boundaries of the spleen are clearly visualized in the horizontal and vertical projections shown in Figs. 3(d) and 3(g). Because the spleen has a fine network of sinusoids and stores a significant amount of blood, it produces a strong and homogeneous PA contrast. The PAT images of the liver show the outline of a lobe and its underlying vascular anatomy in both projections, as shown in Figs. 3(e) and 3(h). The PAT images of the kidney clearly visualize the renal vasculature. The vertical projection in Fig. 3(i) shows the complete longitudinal view of the kidney and its renal vasculature up to 5 mm in depth.

## 4 Conclusion

In summary, a novel dual-modality endoscope that combines PAT imaging and conventional white light videoscopy in a forward-viewing configuration has been presented. High-resolution PAT imaging of biological tissues was demonstrated by visualizing the 3D vascular structures of abdominal organs from an *ex vivo* mouse including spleen, liver, and kidney. In addition, white light images of the tissue surface were acquired, illustrating the dual modal capability of the probe.

The novel aspects of the probe are the use of an MEMs based scanner and optical relay system to read out the FP sensor. The small size and low mass of the MEMs scanner allows the proximal end of the probe to be sufficiently compact and lightweight for practical endoscopic use. This would not be possible using the much bulkier and heavier galvanometer-based scanners used in previous free-space FP sensor read-out schemes.^17^ The relay is also a key enabling design feature. By mapping the optical “pivot point” from the MEMs scanner to the distal end of the probe, it allows the FP sensor to be located at the end of a tube that is sufficiently long (27 cm) for endoscopic use. By contrast, in previous free-space FP scanners, the scanner-sensor distance was much less, typically 5 cm, and thus insufficient for an endoscopic implementation.

The design of the probe offers several inherent advantages. These include the broadband ultrasound frequency response of the FP sensor and fine spatial sampling, which provides high-resolution PAT images and a forward-viewing configuration for surgical guidance applications. The integrated white light imaging capability is useful, not only for providing tissue characterization information but also for navigating to the anatomical area of interest prior to acquiring a PAT image. Moreover, the concept is not limited to integrating white light imaging alone. It would also be possible to integrate other optical imaging techniques, such as narrowband, hyperspectral, fluorescence imaging, or optical coherence tomography.^23^ There is further potential to incorporate a pulse-echo ultrasound imaging capability by depositing a dichroic absorbing coating on to the FP sensor that acts as a laser-generated ultrasound transmitter.^24^

Although feasibility has been demonstrated, translating the concept into practical *in vivo* clinical use will require further development, most obviously to reduce the current PAT acquisition time of 40 s. This could be addressed using a multi-beam scanner^25^ to parallelize the sensor read-out and the use of higher PRF excitation lasers and compressed sensing techniques.^26^ In combination, these approaches offer the prospect of achieving real-time 3D image frame rates.

It is anticipated that this type of probe could ultimately find several clinical endoscopic applications in which a high level of miniaturization is not a primary requirement and a rigid device can be accommodated, for example, in keyhole laparoscopic surgery to remove tumors in the liver and other abdominal organs. Surgical instruments, endoscopes, and rigid laparoscopic ultrasound probes are commonly delivered via ports introduced in the abdominal wall. The diameter of these ports is typically in the 5 to 10 mm range and thus could accommodate our probe, which could be used to visualize the margins of tumors based on their vascular contrast to help guide their removal by surgery or ablative methods.

## Data Availability

Data underlying the results may be obtained from the authors upon request.

## References

[1] Beard P., “Biomedical photoacoustic imaging,” Interface Focus 1(4), 602–631 (2011). 10.1098/rsfs.2011.0028 22866233 PMC3262268

[2] Wang L. V. Yao J., “A practical guide to photoacoustic tomography in the life sciences,” Nat. Methods 13(8), 627 (2016).1548-7091 10.1038/nmeth.3925 27467726 PMC4980387

[3] Wen X. et al., “High-robustness intravascular photoacoustic endoscope with a hermetically sealed opto-sono capsule,” Opt. Express 28(13), 19255–19269 (2020). OPEXFF 1094-4087 10.1364/OE.394781 32672206

[r4] Wang L. et al., “Tapered fiber-based intravascular photoacoustic endoscopy for high-resolution and deep-penetration imaging of lipid-rich plaque,” Opt. Express 27(9), 12832–12840 (2019). OPEXFF 1094-4087 10.1364/OE.27.012832 31052818

[r5] Cao Y. et al., “Fast assessment of lipid content in arteries in vivo by intravascular photoacoustic tomography,” Sci. Rep. 8(1), 2400 (2018). SRCEC3 2045-2322 10.1038/s41598-018-20881-5 29402963 PMC5799328

[r6] Horiguchi A. et al., “Pilot study of prostate cancer angiogenesis imaging using a photoacoustic imaging system,” Urology 108, 212–219 (2017). 10.1016/j.urology.2017.07.008 28735020

[7] Zhu Y. et al., “Prototype endoscopic photoacoustic-ultrasound balloon catheter for characterizing intestinal obstruction,” Biomed. Opt. Express 13(6), 3355–3365 (2022). BOEICL 2156-7085 10.1364/BOE.456672 35781972 PMC9208587

[8] Li G. Guo Z. Chen S-L., “Miniature probe for forward-view wide-field optical-resolution photoacoustic endoscopy,” IEEE Sens. J. 19(3), 909–916 (2018). ISJEAZ 1530-437X 10.1109/JSEN.2018.2878801

[r9] Lu C. et al., “Electrothermal-MEMS-induced nonlinear distortion correction in photoacoustic laparoscopy,” Opt. Express 28(10), 15300–15313 (2020). OPEXFF 1094-4087 10.1364/OE.392493 32403561

[r10] Guo H. et al., “Photoacoustic endoscopy: a progress review,” J. Biophotonics 13(12), e202000217 (2020). 10.1002/jbio.202000217 32935920

[11] Li G. et al., “Miniature probe for dual-modality photoacoustic microscopy and white-light microscopy for image guidance: a prototype toward an endoscope,” J. Biophotonics 13(4), e201960200 (2020). 10.1002/jbio.201960200 31920005

[12] Wang Y. Stephens D. N. O’Donnell M., “Optimizing the beam pattern of a forward-viewing ring-annular ultrasound array for intravascular imaging,” IEEE Trans. Ultrason. Ferroelectr. Freq. Control 49(12), 1652–1664 (2002). ITUCER 0885-3010 10.1109/TUFFC.2002.1159845 12546147

[13] Arkan E. F. Degertekin F. L., “Analysis and design of high-frequency 1-D CMUT imaging arrays in noncollapsed mode,” IEEE Trans. Ultrason. Ferroelectr. Freq. Control 66(2), 382–393 (2019). ITUCER 0885-3010 10.1109/TUFFC.2018.2887043 30571620 PMC6415772

[r14] Nikoozadeh A. et al., “Photoacoustic imaging using a 9F microLinear CMUT ICE catheter,” in IEEE Int. Ultrason. Symp., IEEE, pp. 24–27 (2012). 10.1109/ULTSYM.2012.0007

[r15] Vaithilingam S. et al., “Three-dimensional photoacoustic imaging using a two-dimensional CMUT array,” IEEE Trans. Ultrason. Ferroelectr. Freq. Control 56(11), 2411–2419 (2009). ITUCER 0885-3010 10.1109/TUFFc.2009.1329 19942528

[16] Moini A. et al., “Fully integrated 2D CMUT ring arrays for endoscopic ultrasound,” in IEEE Int. Ultrason. Symp. (IUS), IEEE, pp. 1–4 (2016). 10.1109/ULTSYM.2016.7728542

[17] Zhang E. Laufer J. Beard P., “Backward-mode multiwavelength photoacoustic scanner using a planar Fabry-Perot polymer film ultrasound sensor for high-resolution three-dimensional imaging of biological tissues,” Appl. Opt. 47(4), 561–577 (2008). APOPAI 0003-6935 10.1364/AO.47.000561 18239717

[18] Ansari R. et al., “Miniature all-optical flexible forward-viewing photoacoustic endoscopy probe for surgical guidance,” Opt. Lett. 45(22), 6238–6241 (2020). OPLEDP 0146-9592 10.1364/OL.400295 33186959 PMC8219374

[19] Ansari R. et al., “All-optical forward-viewing photoacoustic probe for high-resolution 3D endoscopy,” Light Sci. Appl. 7(1), 75 (2018). 10.1038/s41377-018-0070-5 30323927 PMC6177463

[20] Ansari R. et al., “Use of a flexible optical fibre bundle to interrogate a Fabry–Perot sensor for photoacoustic imaging,” Opt. Express 27(26), 37886–37899 (2019). OPEXFF 1094-4087 10.1364/OE.27.037886 31878562 PMC7046039

[21] Treeby B. E. Zhang E. Z. Cox B. T., “Photoacoustic tomography in absorbing acoustic media using time reversal,” Inverse Probl. 26(11), 115003 (2010). INPEEY 0266-5611 10.1088/0266-5611/26/11/115003

[22] Beard P. C. Perennes F. Mills T. N., “Transduction mechanisms of the Fabry-Perot polymer film sensing concept for wideband ultrasound detection,” IEEE Trans. Ultrason. Ferroelectr. Freq. Control 46(6), 1575–1582 (1999). ITUCER 0885-3010 10.1109/58.808883 18244356

[23] Chen Z. et al., “Non-invasive multimodal optical coherence and photoacoustic tomography for human skin imaging,” Sci. Rep. 7(1), 17975 (2017). SRCEC3 2045-2322 10.1038/s41598-017-18331-9 29269886 PMC5740114

[24] Pham K. et al., “Broadband all-optical plane-wave ultrasound imaging system based on a Fabry–Perot scanner,” IEEE Trans. Ultrason. Ferroelectr. Freq. Control 68(4), 1007–1016 (2021). ITUCER 0885-3010 10.1109/TUFFC.2020.3028749 33035154

[25] Huynh N. et al., “Photoacoustic imaging using an 8-beam Fabry-Pérot scanner,” Proc. SPIE 9708, 97082L (2016). PSISDG 0277-786X 10.1117/12.2214334

[26] Arridge S. et al., “Accelerated high-resolution photoacoustic tomography via compressed sensing,” Phys. Med. Biol. 61(24), 8908 (2016). PHMBA7 0031-9155 10.1088/1361-6560/61/24/8908 27910824

